# Infertility and physical activity: A cross-sectional study of women living in Yazd aged 20-49 yr, 2014-2015

**DOI:** 10.18502/ijrm.v13i9.7673

**Published:** 2020-09-20

**Authors:** Masoud Mirzaei, Ph.D., Nasim Namiranian, Behnam Bagheri-Fahraji, Somaye Gholami

**Affiliations:** ^1^Research centre of prevention and epidemiology of non-communicable disease, school of public health, shahid sadoughi university of medical sciences, Yazd, Iran.; ^2^Diabetes Research Center, Shahid Sadoughi University of Medical Sciences, Yazd, Iran.

**Keywords:** Women, Infertility, Physical activity, Yazd, Iran

## Abstract

**Background:**

Changes in the state of energy balance owing to changes in physical activity (PA) may affect the reproductive system.

**Objective:**

the aim of this study was to assess the association between PA and infertility of women living in Yazd 2014 - 2015.

**Materials and Methods:**

The study method was analytical cross-sectional on the Yazd Health Study (YaHS) data which was conducted on 10,000 people. We studied 2,611 women (20 and 49 yrs old), living in Greater Yazd area. PA information was collected using a physical activity questionnaire (IPAQ) short form. The standardized instruments were used for anthropometrics measurement.

**Results:**

Totally 135 cases of infertility were documented. The overall prevalence of infertility was 4.73% (95% CI: 3.94-5.59%). The median of PA scores (METs) in women was 746.66 and 25-75% interquartile range was 361.25-1277.25. The relationship according to the Chi-square test between infertility and PA, as categorized variables, was significant (p = 0.015). It showed over 90% of those who suffered from infertility had a low or moderate PA.

**Conclusion:**

The results of our study showed that there is a significant relationship between infertility and PA level in women living in Greater Yazd area. Also, women with infertility had lower activity levels, consistent with most previous studies.

## 1. Introduction

Infertility is the foremost reproductive disorder in developing countries. The lifetime prevalence rate of infertility is about 6.6% - 32.6% (1, 2). Infertility influences whole life with dysthymia, anxiety, and other psychiatric diseases (3). But, in most developing countries, reproductive healthcare is equal with family planning (4). Obesity and overweight are prevalent issues among Iranian women due to physical inactivity and sedentary lifestyle (5-7). The general health and well-being are influences by lifestyle factors such as physical activity (PA) and body mass index (BMI) influence (8-11). The association of PA and female fertility in the general population was not well studied, in contrast to the relatively well-known beneficial effects of regular PA on non-communicable diseases, including the prevention of premature death (12, 13). The results of recent prospective studies about the impact of PA on infertility are controversial (14-16). A recent study has shown that moderate exercise combined with weight loss in fertility treatment of the obese women were effective (17). PA is essential in maintaining energy balance. As well the energy balance deviations because of PA may affect the reproductive system (18). the decreasing insulin resistance and hormonal changes due to PA and weight loss can protect the ovarian function (9).

The purpose of this study was to evaluate the association between PA and infertility in women aged 20-49 year in a large Iranian population.

## 2. Materials and Methods

This analytical cross-sectional study was done on data from the Yazd Health Study (YaHS), 2014-2015 (19). YaHS was conducted on 10,000 residents of Greater Yazd area (20-69 yr). Greater Yazd area includes Yazd city, which is the capital of Yazd province, and three annexed cities—Shahediah, Zarch and Hamidia—and surrounding villages (19).

“The sampling method of this study was population-based, random, and multi-stage-stratified. Two hundred clusters were selected randomly according to city postcodes. The interviewers contacted the assigned addressees and, after explanation of the study protocol, settled a meeting time at their residences” (19).

The inclusion criteria were age 20-49 yr at the time of interview and Yazd resident. The informed consent to participate in the study was taken from all participants. The exclusion criteria were those who were guests and not residents of the recruited addresses. Data were collected using validated questionnaires.

The standardized instruments were used to measure anthropometrics by trained researchers. “The anthropometric indices including weight, height, and waist and hip circumferences were measured according to standard criteria and calibrated measurement tools. BMI was defined based on weight ratio (kg) on height squared (m) and its limits were determined according to the standard criteria (WHO)” (19). The entire data gathering was based on YaHS study protocol. Detail of the study was published elsewhere (19).

The infertility history and its related factors were checked by all women who participated in the study. Infertility was defined as “no pregnancy after one year of unprotected coitus (9), primary infertility was defined as the failure to achieve a clinical pregnancy after 12 months or more unprotected coitus”. The secondary infertility was defined as “the inability to be pregnant after first pregnancy” (20).

PA was measured using a validated Persian translated copy of the original International Physical Activity Questionnaire (IPAQ) short form (21, 22). Data from the IPAQ-SF were classified into three categories: low, moderate, and high PA. This is based on the METs score as defined by the IPAQ working group (23).

Out of the 9,965 participants, 4,989 were female and 2,611 were in the reproductive age (i.e., 20-49 years). In the YaHS study protocol (9), age was considered as the categorical variable; 20-29, 30-39, 40-49, 50-59 and 60-69 yr. However, the participants 20-49 yr were included in our study.

### Ethical consideration

This study was approved by the Ethics Committee of Shahid Sadoughi University of Medical Sciences (number 73941/1/17) on 8/7/2014 and informed consent was obtained from all participants.

### Statistical analysis 

Descriptive statistics were reported as a percentage, mean (standard deviation) Pearson correlation, Chi-square, independent *t* test, and non-parametric equals' analysis with a significant level of 0.05 were used. Also, the infertility prevalence which is reported in this study is age adjusted according to the Yazd Greater Area population age distribution in the 2011 national census.

## 3. Results

Out of the 9,965 participants included in the YaHS study, 4,989 (50.1%) were women aged 50-69 yr, that is, who were not at reproductive age range and were therefore excluded, and only 2,611 women aged 20-49 yr were studied. Table I showed the baseline characteristics of studied sample. The age-adjusted prevalence of infertility (95% confidence interval) was 4.73% (CI = 3.94-5.59) in the 20-49 year population. The median of PA scores (METs) in women aged 20-49 yr in Yazd city in 2012-2013 was 746.66 and 25-75% interquartile range was 361.25-1277.25. The PA scores (METs) normal distribution cure was right skewed and the Shapiro-Wilk test showed that the distribution was not normal.

The relationship according to the Chi-square test between infertility and PA, as categorized variables, was significant (p = 0.015). It showed over 90% of those who suffer from infertility have a low or moderate PA. In contrast, the prevalence of infertility was higher in women with less PA.

The relationship between the cause of infertility, types of infertility, and level of PA was not statistically significant. The results of this review are presented in Table II. The above result was also quantitatively evaluated in the level of PA.

In the analysis (Mann-Whitney U test), the relationship of infertility and PA was quantitatively significant (based on the METs score), the mean of PA score in infertile is significantly lower than the fertile women (p = 0.019). PA level showed a significant correlation with age (p = 0.004), education level (p = 0.001), and waist circumference (p = 0.014). There were no significant relationship between PA with smoking (p = 0.147) and PA with BMI (p = 0.053) (Table III).

Table IV shows the association of PA variables with infertility with controlling for confounding variables including education level, age, BMI, smoking, and waist circumference. The results of logistic regression analysis showed that the variables of smoking and education level were not significant (p > 0.05), but the variables of age, BMI, and waist circumference were significant and remained in the model (p < 0.05).

The chance of infertility in women aged 30-39 is 2.16 times that of women aged 20-29 yr, and the risk of infertility in women aged 40-49 is 2.211 times that of women aged 20-29. They are up to 29 years. The chances of infertility in women whose BMI is below 18.5 are 6.09 times that of women whose BMI is normal and between 18.5 and 24.9. The chances of infertility in women older than40 is 3.44, 2.66, 4.98 for women whose BMI is below normal, and between 18.5 - 24.9 and more than 25. The likelihood of infertility in women with high waist circumference is 14.29 times that of women with normal waist circumference. The chance of infertility in those with low PA is 3.51 times higher than those with moderate PA. The likelihood of infertility in people with high PA is 2.01 times higher than those with moderate PA.

The results showed that BMI and waist circumference had a significant effect on the odds of infertility and the OR between PA and infertility in the presence of BMI and waist circumference was 3.5, which decreases to 1.2 if not included in the model. This indicates that this variable must remain in the model. Interaction between BMI and waist circumference with the PA were not statistically significant.

**Table 1:** Baseline characteristics of YaHS female participants aged 20-49 years in Yazd-Iran


**Variable**		**Frequency (%)**
**Age (yr)**		
	**29-20**	700 (26.8)
	**39-30**	927 (35.5)
	**49-40**	984 (37.7)
**Marital status**		
	**Married**	2530 (96.9)
	**Widow**	54 (2.1)
	**Divorced**	27 (1)
**Education**		
	**Primary school and less**	395 (15.1)
	**High school**	821 (31.5)
	**Diploma and graduate diploma**	955 (36.6)
	**Bachelor of sciences**	394 (15)
	**Master of science and Doctorate**	46 (1.8)
**Body mass index (BMI)**		
	**Lack of weight < 18.5**	87 (3.3)
	**Normal weight 24.9-18.5**	811 (31.1)
	**Overweight 29.9-24.9**	1000 (38.3)
	**Chubby 39.9-29.9**	663 (25.4)
	**Extreme obese > 39.9**	49 (1.9)
**Waist circumference**		
	**Normal / Under 88 cm**	881 (33.8)
	**Obese / Over 88 cm**	1729 (66.2)
**Desire to have a child**		
	**Yes**	607 (23.3)
	**I do not think so**	340 (13)
	**No**	1506 (57.7)
**Positive family history of infertility**		
	**Yes**	484 (73.2)
	**I do not know**	214 (8.3)
	**No**	1912 (18.5)
**Abortion and stillbirth history**		
	**Yes**	603 (24.9)
	**No**	1818 (75.1)

**Table 2:** Relationship of infertility with PA Level in women aged 20-49 years living in Yazd Greater Area during 2014-2015


**Sub-group**		**Low physical activity**	**Moderate physical activity**	**Severe physical activity**	**P-value**
**Cause of infertility**					
	**Female**	34 (0.3)	34 (40.5)	6 (18.2)	
	**Both**	8 (8.1)	11 (13.1)	2 (6.1)	
	**Unexplained**	57 (57.6)	39 (46.4)	35 (75.8)	0.065
**Infertility type**					
	**Primary**	52 (50.5)	39 (46.4)	13 (12.6)		
	**Secondary**	45 (48.4)	38 (40.9)	10 (10.9)	0.824
Data presented as frequency (%); Statistical test: Chi-square					

**Table 3:** The relation of some variables with the level of PA in women aged 20-49 years living in Yazd Greater Area during 2014-2015


**Variable/Infertility**		**Low physical activity**	**Moderate physical activity**	**Severe physical activity**	**P-value**
**Level of education**					
	**Primary school and less**	229 (15.7)	165 (15.4)	66 (12.1)	
	**High school**	440 (30.2)	298 27.8)	123 (22.5)	
	**Diploma and graduate diploma**	519 (35.7)	392 (36.6)	123 (22.5)	
	**B.Sc.**	237 (16.3)	194 (18.1)	118 (21.6)	
	**M.Sc. and Doctorate**	30 (2.1)	23 (2.1)	28 (5.1)	0.001
**Age range (yr)**					
	**20-29**	481 (33.1)	324 (30.2)	195 (35.7)		
	**30-39**	473 (32.5)	362 (33.8)	193 (35.3)		
	**40-49**	501 (34.4)	386 (36)	158 (28.9)		0.004
**Body mass index**						
	**< 18.5**	45 (3.3)	23 (2.1)	24 (4.6)		
	**18.5-24.9**	444 (32.2)	331 (32.1)	184 (35.6)	
	**25-29.9**	448 (32.4)	357 (34.6)	172 (33.3)	
	**30-39.9**	371 (36.9)	262 (25.4)	122 (23.6)	
	**≥ 40**	73 (36.9)	34 (3.3)	15 (2.9)	0.053
Smoking${}^{*}$					
	**Yes**	7 (0.5)	8 (0.7)	5 (0.9)		
	**Sometimes**	17 (0.2)	8 (0.7)	4 (0.7)		
	**Quitted**	6 (0.4)	0 (0)	4 (0.7)		
	**No**	1425 (97.9)	1056 (98.5)	533 (97.6)	0.147
**Waist circumference**					
	**Normal ( < 88 cm)**	522 (36.6)	331 (31.3)	202 (37.1)		
	**High ( < 88 cm)**	917 (63.7)	728 (68.7)	342 (62.9)		0.014
Data presented as frequency (%); *Smoking: No, never smoked; Quitted, a person who has been smoking for some time but is not currently smoking; Sometimes, occasional smoking; Yes, current smoking; Statistical Test: Chi-square						

**Table 4:** Relationship between PA and control of confounding variables in women aged 20-49 years living in Yazd Greater Area during 2014-2015


**Variable/Infertility**	**Subgroup**	**SE**	**Wald**	**OR (adjust)**	**P-value**
	**Primary school and less**	2.54	0.6
	**High school**	0.23	0.35	1.003	099
	**Diploma and graduate diploma**	0.22	0.30	1.13	0.58
	**Bachelor of science**	0.27	1.2	1.34	0.27
**Level of education**	**Master of science and doctorate**	0.61	0.92	1.81	0.33
		**20-29**	14.32	0.001
		**30-39**	0.21	12.7	2.16	0.003
	**Age range (yr)**	**40-49**	0.17	13.89	2.21	0.001
			**< 18.5**	0.64	7.9	6.09	0.001
		**18.5-24.9**	21.64	0.005
	**25-29.9**	0.30	16.5	3.44	0.001
	**30-39.9**	0.29	10.98	2.66	0.001
**Body mass index**	**> = 40**	0.29	15.36	4.98	0.02
		**No**	0.64	0.8
		**Sometimes**	1.04	0.15	1.5	0.6
		**Quitted**	1.2	0.12	1.7	0.9
	**Smoking**	**Yes**	0.74	0.59	1.87	0.44
		**Normal ( < 88 cm)**	34.5	0.001
**Waist circumference**		**High ( > 88 cm)**	0.12	45.6	14.29	0.001
			**Low physical activity**	0.26	6.27	3.51	0.002
	**Moderate physical activity**	8.17	0.017
**PA**	**Severe physical activity**	0.29	8.05	2.01	0.005
	PA*Waist circumference	0.247	0.6	0.826	0.439
	PA*BMI	0.168	0.7	1.15	0.403
Statistical test: Logistic regression; ${}^{*}$Relationship between PA and infertility after adjusting for age, waist circumference, BMI and education level					

## 4. Discussion

In this study, the relation between PA and infertility in women aged 20-49 yr who lives in Yazd greater area were examined. It was found that the prevalence of infertility was higher in women with the low frequency or intensity of PA. The association of PA with some of the variables studied in women aged 20-49 yr was evaluated. Infertility relationship was significant with education level, age, BMI, and waist circumference.

PA is one of the main modifiable risk factor of non-communicable diseases. PA contributes the regulation of blood pressure (24), body weight (25), and improves glucose impairment (26, 27). Some studies stated that PA influences the reproductive system (28-30). Human *et al.* revealed that age, weight, and smoking were correlated with general health and adversely affect reproductive system. However, more studies are needed focusing specifically on the relationship between diet and different PA levelon reproductive performance (31).

In the present study, the association between PA and infertility was established. The mean score of PA was significantly lower in infertile women.

After analyzing this relationship in the logistic regression model and controlling for confounding variables, the results were interesting. While low PA increases the chance of infertility, high PA can also increase the chance of infertility.

The chance of infertility in those with low PA is 3.51 times higher than those with moderate PA. The likelihood of infertility in people with high PA is 2.01 times higher than those with moderate PA.

Rich-Edwards and colleagues study concluded that exercise was associated with reduced risk of ovulatory infertility (16). After BMI adjustment, each hour of vigorous exercise per week was related with 5% risk reduction of infertility. PA may protect ovarian function independent of BMI. In the present study it was found that, the chances of infertility in women whose BMI is below 18.5 are 6.09 times that of women whose BMI is normal and between 18.5 and 24.9. Above 40 is 3.44, 2.66, 4.98 for women whose BMI is normal, and between 18.5 and 24.9. The likelihood of infertility in women with high waist circumference is 14.29 times that of women with normal waist circumference.

The present study was conducted to investigate the modifier effect of BMI and waist circumference on the association between PA and infertility. As shown in Table IV, interaction between BMI and waist circumference with the PA were not statistically significant.

But, no association with moderate exercise was seen. The mechanisms which shows the intensive PA as the risk factor of infertility are not entirely understood and may intensive PA disrupts ovulation (32, 33). In this study, we were able to demonstrate a significant association between PA and infertility. Recent study has shown that PA improves ovarian function through insulin sensitivity (34). Insulin sensitivity can act at the ovarian level, reducing the response to the gonadotrophin stimulation (35). However, the results of recent studies have often been performed on PCOS and PA has been effective in reducing infertility due to weight loss (35, 34). Endometriosis is one of the factors affecting infertility in women (31-33). According to a meta-study by Ricci *et al*., PA can reduce the risk of infertility (34). This can be one of the reasons for the impact of PA on infertility in the current research. According to a meta-analysis study by Gabriela *et al*., PA intervention in previous RCT studies has been able to improve fertility rates in women with infertility disorders (36).

### Limitation

Our research has some limitations; first of all infertility was evaluated by a questionnaire, not specialist diagnosis. But in most of large epidemiologic studied this limitation is repeated. The age range of studied population is the other limitation. According to the National Portal of Statistics of Iran in 2015, 25.2% of women under the age of 20 were married [14-19 yr old] who were not considered in this study. The main strong point of the study was random sampling method and great sample size (37). Also, the interviewers were well-trained for interview and anthropometrics measurements were done with calibrated instruments.

## 5. Conclusion

Our study reported that there is a significant relationship between infertility and PA level in women living in Yazd Greater Area. Also, women with infertility had lower activity levels, consistent with most previous studies. The level of PA was significantly related to age, level of education, and waist circumference.

##  Conflict of Interest

The authors declare no conflict of interest.
